# A Unique Case Report of Rhabdomyosarcoma Arising Within a Sacrococcygeal Teratoma

**DOI:** 10.7759/cureus.107324

**Published:** 2026-04-19

**Authors:** Jenusha Felix, Jyotsna Yesodharan, Naveen Viswanath

**Affiliations:** 1 Pathology, Amrita Institute of Medical Sciences, Kochi, IND; 2 Pediatric Surgery, Amrita Institute of Medical Sciences, Kochi, IND

**Keywords:** paediatric case, recurrence, rhabdomyosarcoma, sacrococcygeal teratoma, somatic-type malignancy

## Abstract

Teratomas are germ cell tumours* *(GCTs) composed of derivatives from one or more of the three germ layers. Sacrococcygeal teratomas (SCTs) are common in the early paediatric age group, with surgical excision being the primary mode of treatment. Malignancy in the context of teratomas occurs either as an immature teratoma or, rarely, as somatic malignancies arising from a germline component within the teratoma. Here, we report an extremely rare case, initially diagnosed as presacral rhabdomyosarcoma, which was subsequently found to have arisen within an SCT in a two-year-old child. To the best of our knowledge, we could not identify a similar case in English literature, and this highlights the need for publication to enable standardisation of management protocols for these extremely rare conditions.

## Introduction

The word teratoma arises from the Greek words ‘teras-’ and ‘-oma’, meaning monster and tumour, respectively, attributed to the presence of hair and teeth in the gross specimens [1]. These tumours originate from totipotent germ cells that fail to migrate during growth and development of the foetus, thereby seen more commonly in the midline in neonates. Teratomas usually arise in the gonads in the young adult age group.

Teratomas have been traditionally classified into mature or immature based on their histological composition. Mature teratomas, with mature tissue components, are considered benign, while immature teratomas bearing immature components are potentially malignant. They may also coexist with other malignant germ cell tumours (GCTs) or, rarely, harbour a somatic-type malignancy like a carcinoma or sarcoma [2]. In a case report by Fein and Hobart, they refer to prior reports of malignant transformation in teratomas, almost all of which were carcinomas [3]. However, the nomenclature for this entity has evolved from teratoma with malignant component in the 1970s, to Teratoma with Somatic Type Malignancy (TSMT) in the 2004 WHO Blue books [4].

Although our review of published English literature showed a few case reports of rhabdomyosarcoma arising within retroperitoneal, gonadal and mediastinal teratomas, its association with a sacrococcygeal teratoma (SCT) has not been previously reported.

## Case presentation

Our patient, a two-year-old female child, initially presented to the local hospital with complaints of constipation for the past three weeks and abdominal pain of three days' duration. Ultrasonography of the abdomen showed a retroperitoneal/presacral solid mass. Magnetic resonance imaging was suggestive of SCT. She was referred to the Department of Paediatric Surgery at our hospital for further evaluation and management.

**Figure 1:**
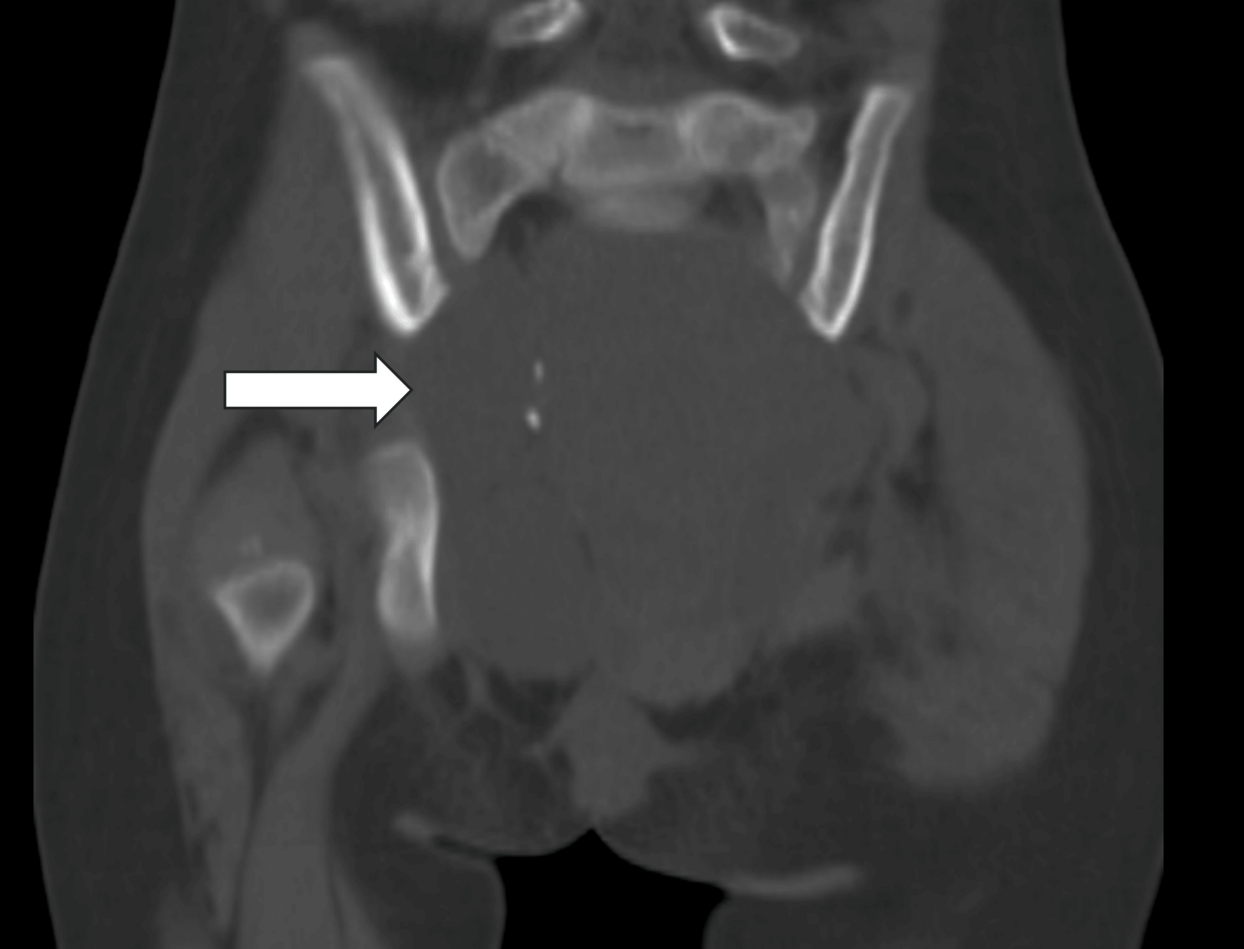
CT image. Complex solid lesion with few cystic components and a few calcific foci seen in the pelvis, in the pre-sacral region.

On examination, she had a palpable pelvic mass; other systems were within normal limits. A computed tomography (CT) scan of the pelvis showed a presacral complex solid lesion measuring 7.6 × 6.5 cm with cystic components, fat and calcific foci. Inferiorly, the lesion was seen reaching the anal verge, perineum and ischio-anal fossa, displacing the sigmoid colon and rectum anteriorly. No intraspinal extension or bone destruction was seen. A 7 × 8 cm exophytic component was noted arising from the primary lesion (Figure 1).

**Figure 2:**
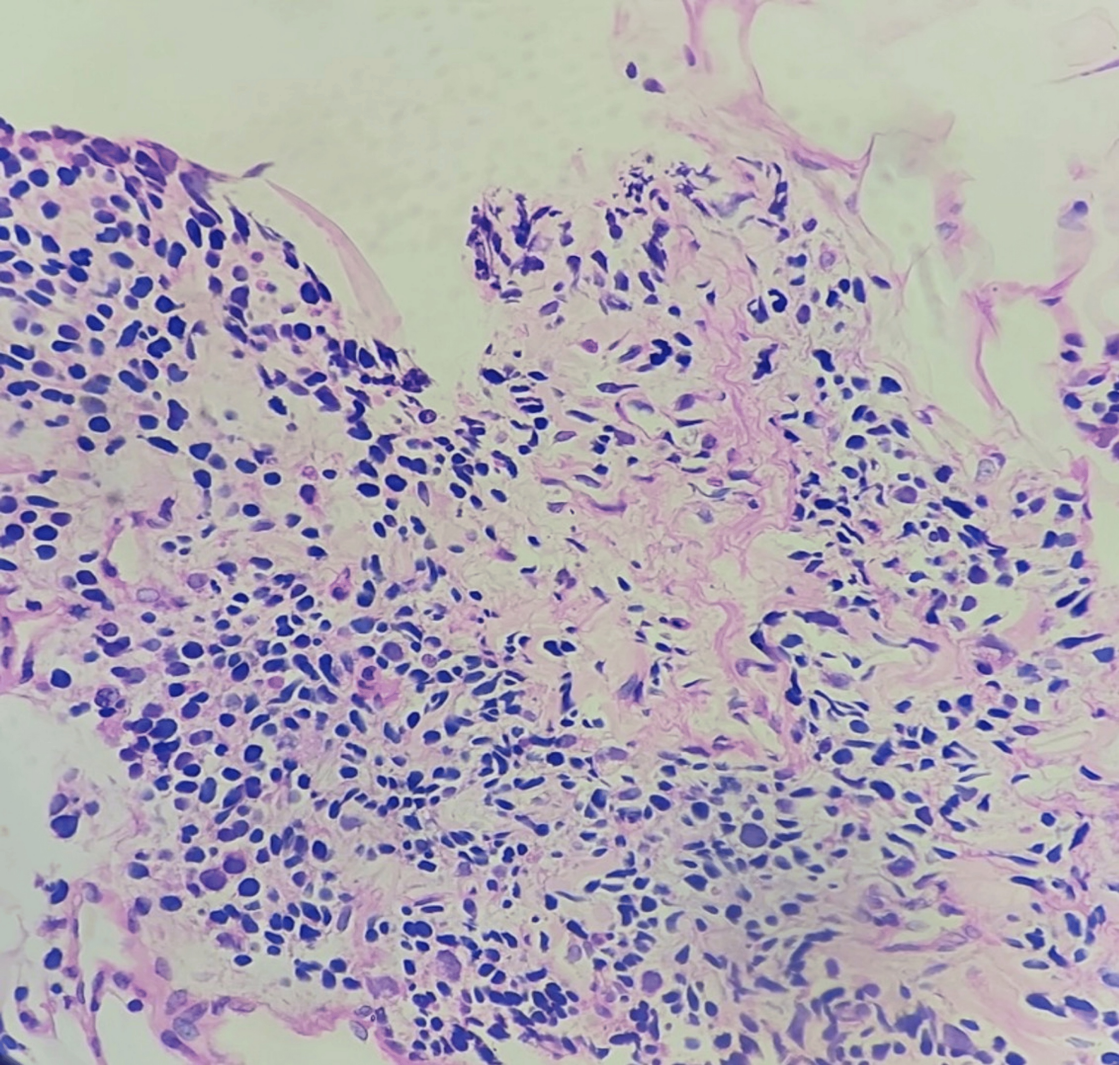
Cells with enlarged eccentrically placed nuclei with smudgy chromatin with moderately dense eosinophilic cytoplasm at 400× magnification.

**Figure 3:**
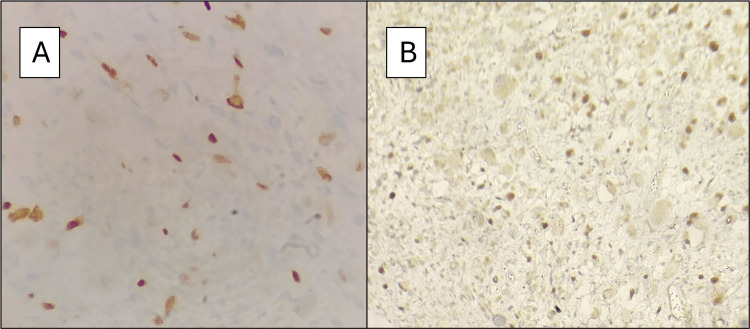
Immunohistochemistry. (A) Cells expressing MyoD1 positivity at 400× magnification. (B) Cells expressing MyoD1 positivity at 100× magnification.

The child was treated symptomatically with laxatives and scheduled for excision of the presacral mass. However, intra-operative frozen section evaluation of core biopsy from the mass revealed a small round cell neoplasm suspicious of Rhabdomyosarcoma; hence, excision was deferred. The formalin-fixed, paraffin-embedded histopathology sections showed a variably cellular neoplasm composed of small round cells arranged in diffuse sheets. Cells with eccentrically placed nuclei exhibiting nuclear atypia, smudgy chromatin and dense eosinophilic cytoplasm were also noted (Figure 2), along with mitotic figures and apoptotic bodies. On immunohistochemistry, the cells expressed Desmin and Myf4 (Figure 3), with non-specific CD99; pan CK and S 100 were negative - findings suggestive of rhabdomyosarcoma. 

She was started on neo-adjuvant chemotherapy with Intergroup Rhabdomyosarcoma Study Group IV (IRS-IV) protocol. Interval imaging demonstrated a significant reduction in the size of the lesion, following which she underwent excision.

**Figure 4:**
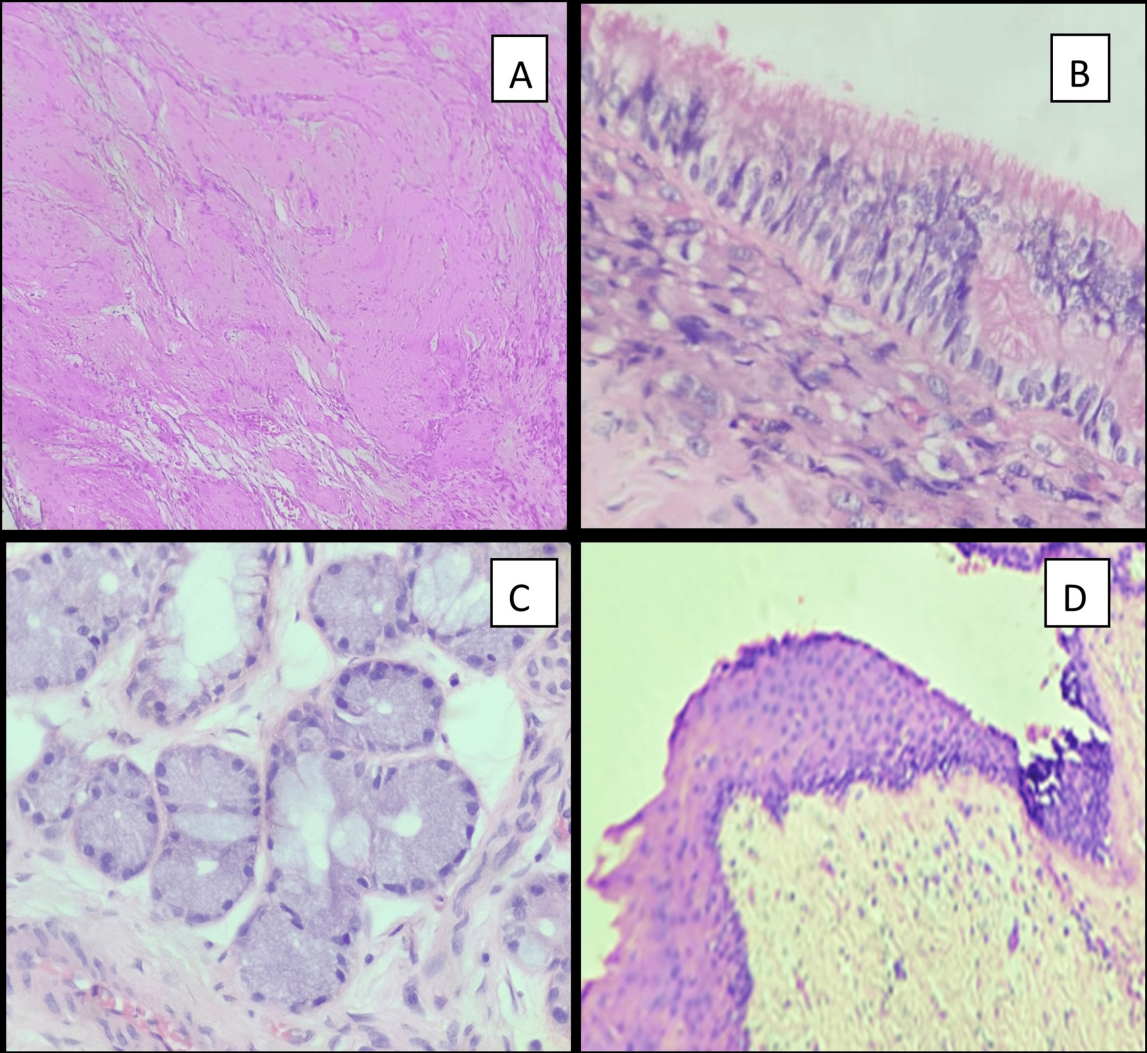
(A) Cyst wall containing glial elements at 100× magnification. (B) Cyst wall with respiratory epithelium at 400× magnification. (C) Cyst wall with seroumucinous glands at 400× magnification. (D) Cyst wall with stratified squamous lining at 400× magnification.

**Figure 5:**
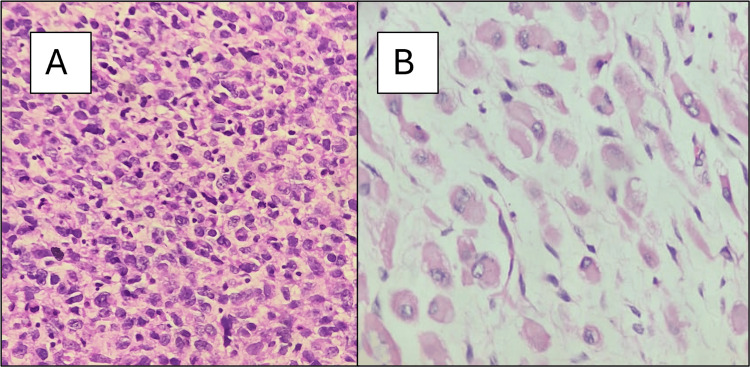
Post-chemotherapy excision specimen. (A) Immature rhabdoid cells at 400× magnification. (B) Mature rhabdoid cells at 400× magnification.

The gross specimen received for histopathology examination consisted of a 5 cm nodular mass with myxoid and cystic areas. Microscopy revealed a variably cellular neoplasm composed predominantly of large cells with eccentrically placed nuclei with smudgy chromatin, prominent nucleoli and moderate to abundant dense eosinophilic cytoplasm. Multinucleated and bizarre cells were seen. Areas with neoplastic cells having pleomorphic ovoid to spindle-shaped, wavy, hyperchromatic nuclei and scant to moderate cytoplasm were also seen. In addition, skin with appendages (Figure 4D), seromucinous glands (Figure 4C), cystic spaces lined by cuboidal/pseudostratified ciliated columnar epithelium (Figure 4B), adipocytes, Schwannian stroma (Figure 4A), calcification and myxoid changes were noted. A diagnosis of rhabdomyosarcoma with post-therapy differentiation arising within a teratoma (teratoma with associated somatic-type malignancy) was offered. Rhabdomyosarcomatous areas (Figure 5A) constituted 60-65% of the sampled tumour, with 85-90% therapy-induced cytodifferentiation (Figure 5B). She underwent adjuvant chemotherapy and, at 10 months post-surgery, was in complete clinical remission.

However, four months later, she presented with pain in her buttocks and constipation. Repeat imaging revealed a large, heterogeneously enhancing solid lesion in the right presacral space, measuring 6.5 × 5.5 × 5 cm. She underwent tumour re-excision. Histopathology findings were consistent with recurrent rhabdomyosarcoma, with approximately 85% viable tumour. She was planned for post-operative radiotherapy. However, three weeks after surgery, she developed a mass in the right gluteal region. This time, imaging showed an increase in size of the presacral mass with pressure symptoms and infiltration. The final agreed plan of management in the tumour board was second-line treatment with high-dose chemotherapy, allogenic stem cell transplant on achieving remission or palliative radiotherapy if symptomatic.

## Discussion

Teratoma is a common GCT composed of tissue derived from two to three germ layers. Teratomas can arise at various anatomical locations, including intracranial, head and neck, mediastinal, retroperitoneal and sacrococcygeal regions, possibly due to the disturbed migration of primordial germ cells. The sacrococcygeal region is the most frequent extragonadal site of involvement by GCTs in children [5].

Although teratoma is the most common histological subtype of GCT reported in sacrococcygeal location, it is rare, with an incidence of 3.7-7.1 cases per 100,000 live births and a clear female predominance [5]. They usually present as antenatal or neonatal masses detected by early ultrasonography [6]. Altman classified SCTs into four classes based on their location relative to the sacral bone. The most common (45%) type I tumours are predominantly external. Type II tumours present externally with a considerable intrapelvic portion, type III is mainly intrapelvic (10%), while type IV is presacral without an external component (10%). Large types II-IV SCT can exert mass effects on intrapelvic organs and present with constipation, faecal incontinence, neurogenic bladder and urinary incontinence.

As per the latest WHO classification [7], teratomas of the testis are classified into pre-pubertal and post-pubertal, including those with somatic type malignancy, wherein all post-pubertal teratomas are considered malignant. Ovarian teratomas are classified into benign mature teratomas with mature tissue elements and malignant immature teratomas, graded based on the amount of immature/neural elements found on microscopy. Mixed GCTs include neoplasms with at least two different GCT components and often include teratomas, mature or immature, with any other benign or malignant counterpart - the commoner ones being yolk sac tumours and embryonal carcinomas.

Malignant GCTs account for about 3-4% of malignancies in children [6]. The 2004 edition of the WHO classification of tumours, termed teratoma with somatic type malignancy as a separate entity, defining it as a teratoma containing a malignant component of a type typically encountered in other organs and tissues, reported ones being rhabdomyosarcoma, undifferentiated sarcoma, squamous cell carcinoma and adenocarcinoma. However, the amount of somatic elements necessary to make this diagnosis remains under discussion.

While the incidence of malignancy in SCTs is approximately 10% in neonates, it approaches universality by three years of age [8]. Mixed GCT with a yolk sac tumour is more common in SCTs than somatic type malignancies [6], which are mostly reported in the gonadal regions. On an extensive search of the literature, we came across rare cases of malignancy, including neuroblastomas and carcinomas arising within SCTs. However, there are no definite clinical or radiological signs to predict malignant transformation within a teratoma [9].

## Conclusions

In the present case, based on the initial biopsy diagnosis of rhabdomyosarcoma, the patient was started on neoadjuvant chemotherapy. It was only upon excision of the lesion that the diagnosis of teratoma with somatic type malignancy, rhabdomyosarcoma, was made, due to the presence of derivatives from different germ cell layers in the resected specimen. Although the primary mode of treatment of teratoma is surgical excision, there is limited literature on the treatment and prognosis of tumours containing somatic-type malignancy due to their rarity. The current strategy is to target the malignant component by either complete surgical resection of the tumour or resection of the residual disease after systemic therapy optimized for histology. The role of adjuvant chemotherapy remains unclear. While GCT-directed chemotherapy benefits only a malignant GCT, radiotherapy warrants consideration when the somatic malignancy is a sarcoma.

In view of their extreme rarity, large-scale multi-institutional studies are warranted to enable data collection and compilation to help streamline management protocols for such patients.
